# Polyphenols from *Broussonetia papyrifera* Induce Apoptosis of HepG2 Cells via Inactivation of ERK and AKT Signaling Pathways

**DOI:** 10.1155/2021/8841706

**Published:** 2021-03-23

**Authors:** Chen-Zhuo Dou, Yan-Fen Liu, Lu-Lu Zhang, Shao-Hong Chen, Chuan-Yin Hu, You Liu, Yun-Tao Zhao

**Affiliations:** ^1^College of Coastal Agricultural Sciences, Guangdong Ocean University, Zhanjiang, Guangdong 524088, China; ^2^Institute of Psychiatry and Neuroscience, Xinxiang Medical University, Xinxiang, Henan 453003, China; ^3^Department of Bioengineering, College of Food Science, Guangdong Ocean University, Zhanjiang, Guangdong 524088, China; ^4^Department of Biology, Guangdong Medical University, Zhanjiang 524088, Guangdong, China

## Abstract

The extract of *Broussonetia papyrifera* has been proved to have antitumor activity. However, the underlying mechanism remains unclear. This study aimed to elucidate the mechanism of apoptosis of HepG2 cells induced by polyphenols from *Broussonetia papyrifera* (PBPs). The results revealed that PBPs inhibited the proliferation of HepG2 cells in a dose-dependent and time-dependent manner. Flow cytometry analysis showed that PBPs increased the apoptosis ratio of HepG2 cells significantly. PBPs increased intracellular reactive oxygen species (ROS) production and decreased intracellular superoxide dismutase (SOD) level of HepG2 cells. PBPs induced cell cycle arrest at G1 phase. Western blotting showed that PBPs upregulated the ratio of *Bax*/*Bcl*-2 and the expression level of *Caspase*-3, and activated *p*53 in HepG2 cells. The inhibition of proliferative relative signals (protein kinase B, PKB/AKT) and survival relative signals (extracellular signal-regulated kinase, ERK) were also observed in PBP-treated HepG2 cells. Our findings suggest that apoptosis of HepG2 cells induced by PBPs is mitochondria-mediated via inactivation of ERK and AKT signaling pathways.

## 1. Introduction

Hepatocellular carcinoma (HCC) is the most common primary malignancy of liver, which results in a heavy medical burden worldwide. Additionally, HCC is estimated to be the second most frequent cause of cancer-related death [1]. The incidence and mortality of HCC have been increasing in North America and several European regions, yet declining in traditionally high-risk countries and regions, including Japan and parts of China [2, 3]. Prognosis of HCC remains poor, with a 5-year survival rate of just 18%. When detected early, HCC patients are amenable to surgical resection; however, the recurrence rate 5 years after resection is about 70% [4]. Nowadays, the most widely used target-specific modern synthetic chemotherapeutic drugs frequently invoke side effects [5, 6]. Therefore, it is of great concern that the effective and safe agents are developed for the prevention and treatment of HCC.

Natural products have been increasingly used as major sources and lead compounds in drug development. Natural phytochemicals used for the cancer treatment are becoming important for drug discovery and research [7]. The use of herbal medicines as alternatives is popular in China and throughout the world [8]. *Broussonetia papyrifera*, a deciduous tree, grows naturally in Asia and Pacific Rim countries such as China and Thailand [9]. Its roots, barks, and fruits have been used as traditional Chinese medicine since ancient times. Bioactive components extracted from *Broussonetia papyrifera* have antibacterial [10], antioxidant [11], anti-nociceptive, anti-inflammatory [12], and antitumor [13] activities. The extract of *Broussonetia papyrifera* leaves exhibits cytotoxicity towards HepG2 cells [14]. Meanwhile, the *n*-butanol fraction of *Broussonetia papyrifera* inhibits the proliferation of colon cancer cell line HT-29 cells [15]. However, the apoptosis mechanism of HepG2 cells induced by the extract of *Broussonetia papyrifera* is still not fully understood.

In this study, the polyphenols from *Broussonetia papyrifera* (PBPs) were prepared, and the effects of PBPs on proliferation and apoptosis of HepG2 cells were studied. Meanwhile, the underlying mechanism was investigated.

## 2. Materials and Methods

### 2.1. Chemicals and Materials

Formic acid (HPLC grade) and acetonitrile (HPLC grade) were purchased from Fisher (Waltham, Massachusetts, USA). Dulbecco's Modified Eagle Medium (DMEM) was acquired from HyClone Laboratories. Fetal bovine serum (FBS) was purchased from Sigma, so were 3-(4,5-dimethylthiazol-2-yl)-2,5-diphenyltetrazolium bromide (MTT), propidium iodide (PI), and 2,7-dichlorofluorescin diacetate (DCFH-DA). Annexin FITC/PI Apoptosis Detection Kit was procured from MultiSciences Biotech Co., Ltd. (Hangzhou, Zhejiang, China). Total SOD Detection Kit was purchased from Nanjing Jiancheng Bioengineering Institute (Nanjing, Jiangsu, China). M-MLV was obtained from Promega Corporation, and SYBR^R^ Premix Taq™ from TaKaRa. Protease/phosphatase inhibitor cocktail was purchased from Roche (USA). Antibodies specific for *Bax*, *Bcl*-2, *Caspase*-3, and *p*53 were procured from Santa Cruz. ERK, p-ERK, AKT, p-AKT, *β*-actin, and HRP-conjugated secondary antibody were purchased from Cell Signaling Technology.

### 2.2. Preparation of PBPs

Fresh bark of *Broussonetia papyrifera* was collected in the nursery of Guangdong Ocean University (Zhanjiang, Guangdong, China). Appropriate dry power was extracted by 65% ethanol solution according to the previous report [16], and the optimal solid-liquid ratio was 1 : 30. The purification was conducted as follows: the phenol-dissolved liquid was concentrated enough with a rotary evaporator (RE-2000A, Shanghai Yarong Biochemical Instrument Plant, Shanghai, China) before the final concentration of 75% ethanol liquid was added. AB-8 macroporous resin (Lu Kang Record Pharmaceutical Co., Ltd., Shandong, China) column was utilized to purify the crude extract. Afterwards, the concentrated liquid was dialyzed in a dialysis bag with 1000 molecular weight cutoff (MWCO 1000, Shanghai Toscience Biotechnology Co., Ltd., Shanghai, China) and lyophilized in a freeze dryer (ES2k, VIRTIS, USA). The quantification of the relative abundance of the isolated phenolic phytochemicals was carried out through UPLC analysis, which was performed on the AcquityTM ultra HPLC system (Waters, USA), and an Agilent ZORBAX-SB C18 (100 mm × 4.6 mm id, 1.8 *μ*m) was used for all separations. The mobile phase, composed of eluent A 0.1% formic and eluent B 0.1% formic acetonitrile solution, was applied in the following gradient elution program: 0 min, 95% A, 30 min, 70% A, 40 min, 5% A, and 41 min, 95% A. The separation temperature was kept constant at 30°C, flow rate and sample volume were set to 0.8 mL/min and 3 *μ*L, and all separations were monitored at 254 nm [11]. The retention time of different compounds (*t*_*R*_) was recorded. To confirm the peaks, the LC-MS experiment using Triple TOF 5600 and a mass spectrometer (AB SCIEX, USA) were performed. The best results were attained when the electrospray in negative ion mode and mass range were set as follows: *m*/*z* 100–1500; GS1, 50 lbf/in2; GS2, 50 lbf/in2; CUR, 35 lbf/in2; TEM, 550°C; IS, −4500 V.

### 2.3. Cell Culture and Treatment

HepG2 cells were provided by Guangdong Medical University Affiliated Hospital (Zhanjiang, Guangdong, China) and were cultured in DMEM supplemented with 10% FBS at 37°C in 5% CO_2_ humidified atmosphere. PBPs were dissolved in DMEM medium, and then the medium was sterilized with a 0.22 *μ*m membrane filter. The working medium was freshly prepared by diluting the appropriate volume of stock solution with the basal medium containing 10% FBS.

### 2.4. Cell Viability Assay

The cell viability was assessed by MTT assay. In brief, 1 × 104 cells/well were seeded into a 96-well plate. After the complete adhesion, PBPs were, respectively, tested at the concentrations of 62.5, 125, 250, and 500 *μ*g/mL for 6 h, 12 h, and 24 h. The cells were then stained with 20 *μ*L MTT (5 mg/mL) for 4 h at 37°C in 5% CO_2_ humidified atmosphere. The supernatant was discarded and 150 *μ*L of DMSO was added and mixed gently for 10 min. The absorbance at 570 nm was measured with a microplate reader (ELx808, Bio Tek, USA).

### 2.5. Apoptosis Assay

HepG2 cells were seeded in 6-well plates (5 × 105 cells/well) and treated with PBPs at different concentrations of 62.5, 125, and 250 *μ*g/mL for 24 h. Then cells were collected, washed twice with ice-cold phosphate buffer saline (PBS, pH 7.4), and stained with fluorescein isothiocyanate (FITC) and PI solution for 10 min in the dark according to the instruction of Annexin FITC/PI Apoptosis Detection Kit. Finally, the apoptotic cells were determined using a flow cytometer (BD FACSCanto™II, Becton Dikson, USA).

### 2.6. Cell Cycle Analysis

HepG2 cells were seeded in 6-well plates (5 × 105 cells/well) and treated with PBPs at concentrations of 62.5 and 125 *μ*g/mL for 24 h. Cells were harvested, washed with ice-cold PBS, suspended with 3 mL 70% ethanol, and placed at −20°C overnight. Then, the fixed cells were stained with 0.25 *μ*g/mL PI solution for 30 min on ice before the flow cytometry according to the previous study [17].

### 2.7. Measurement of Intracellular ROS

The intracellular ROS production was monitored using an oxidant sensitive fluorescent probe DCFH-DA. Briefly, HepG2 cells were seeded in 6-well plates (5 × 105 cells/well) and treated with PBPs at concentrations ranging from 62.5 to 250 *μ*g/mL for 6 h. The cells were harvested and stained with 10 *μ*m DCFH-DA for 30 min at 37°C in 5% CO_2_ humidified atmosphere. The intracellular ROS production was detected by flow cytometry.

### 2.8. Measurement of Intracellular Total SOD Activity

HepG2 cells were seeded in 6-well plates (5 × 105 cells/well) and treated with PBPs at concentrations ranging from 62.5 to 250 *μ*g/mL for 24 h. The cells were harvested and resuspended in water, and then the samples were frozen in liquid nitrogen and thawed at room temperature repeatedly for three times to get supernatant for the analysis of intracellular SOD activity according to the kit instruction. The absorbance was measured at a wavelength of 550 nm by a microplate reader (ELx808, Bio Tek, USA).

### 2.9. Total RNA Isolation and Quantitative Real-Time PCR Analysis

HepG2 cells were cultured in 6-well plates and exposed to PBPs at 100 *μ*g/mL for 12 h. Total RNA was extracted with Trizol reagent. Concentration of the total RNA was determined by the SmartSpec Plus spectrophotometer (Bio-Rad, USA), and the integrity was visualized on 1% agarose gel. The first-strand cDNA was synthesized from 1 *μ*g of total RNA using M-MLV reverse transcriptase and oligo (dT) 18 primers according to the manufacturer's protocol. According to the instruction, 1 *μ*L of template cDNA was added to the final volume of 12.5 *μ*L of reaction mixture, and the parameters of real-time PCR were as follows: 1 min at 95°C followed by 40 cycles involving denaturation at 95°C for 20 s, annealing at 60°C for 20 s, and elongation at 72°C for 20 s. The sequences of the specific sets of primers for *bax*, *bcl*-2, *p*53 [18] and *c-Myc*, *caspase*-3, *β*-actin [19] in this investigation are shown in Table 1. The expression level of each gene was normalized to that of the *β*-actin gene and calculated with the 2^−ΔΔCt^ method. All the real-time PCR experiments were performed in triplicate, and data were expressed as the mean of at least three independent experiments.

### 2.10. Protein Extraction and Western Blotting

HepG2 cells treated with PBPs at different concentrations were harvested after an incubation of 24 h. Being washed with ice-cold PBS, the cells were collected and lysed on ice for 30 min in a modiﬁed RIPA cell lysis buffer containing 50 mM Tris–HCl (pH 7.4), 150 mM NaCl, 1 mM EDTA, 1% NP-40, 0.1% SDS, 0.25% sodium deoxycholate, and protease/phosphatase inhibitor cocktail. The whole cell lysates were clariﬁed by centrifugation at 12,000 rpm at 4°C for 15 min, and protein concentrations were determined by BCA assay. Total proteins were heated for 5 min at 98°C, separated by 12% SDS-polyacrylamide gel electrophoresis (SDS-PAGE), and transferred onto a polyvinylidene diﬂuoride (PVDF) membrane (Millipore, USA). After being blocked in 5% skim milk for 1 h at room temperature, membranes were incubated overnight at 4°C with the speciﬁc primary antibodies. The membranes were washed with PBS/0.1% Tween-20 solution and then incubated with peroxidase-conjugated secondary antibodies (dilution 1 : 2000), washed again, and developed using an enhanced chemiluminescence kit (ECL, Millipore). The protein bands were visualized with the ChemiDoc imaging system (Bio-Rad, USA).

### 2.11. Data and Statistical Analysis

Data were presented as mean ± SD. To analyze the difference between the means of the treatment group and the control group, the one-way ANOVA was applied to calculate the statistical significance. The difference with a *P* value of less than 0.05 was considered statistically significant. All statistics were performed with GraphPad Prism 5.0 (GraphPad, San Diego, CA, USA).

## 3. Results

### 3.1. PBP Constituents

**Figure 1:**
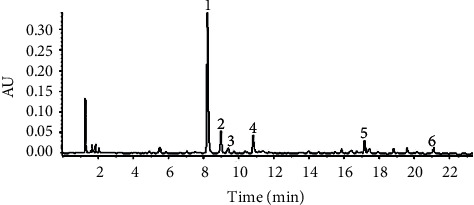
Chromatogram of UPLC analysis of the extract of *Broussonetia papyrifera*. The extract was dissolved in 80% methanol by sonication before analysis. All separations were monitored at 254 nm.

**Figure 2:**
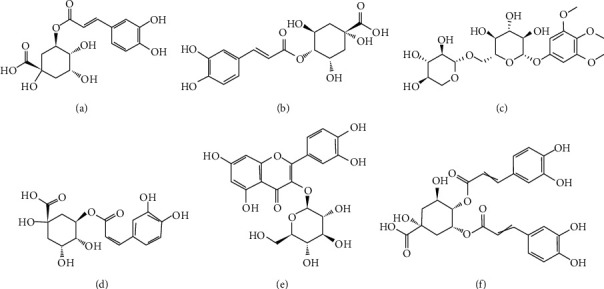
Phenolic phytochemicals isolated from ethanol extractions of *Broussonetia papyrifera* bark: (a) chlorogenic acid; (b) cryptochlorogenic acid; (c) 3, 4, 5-trimethoxyphenyl-1-O-*β*-D-xylopyranosyl-*x*-D-glucopyranoside; (d) cis-form-5-coffee acylchlorogenic acid; (e) isoquercetin; (f) 4, 5-dicaffeoylquinic acid.

As presented in Figure 1, the chromatogram of UPLC showed the variations and the relative quantity of each ingredient. The determination and constituent analysis of PBPs were confirmed by LC-MS/MS. The main components were found to be chlorogenic acid and dicaffeoylquinic acid. The concentration of total phenols in the extract was determined by the relative peak area in the UPLC chromatogram. The structure identification of these compounds was conducted through spectroscopic analyses and by comparison with published data. All fractions of the ethanol extract of *Broussonetia papyrifera* yielded phenolic phytochemicals which were purified by macroporous resin as delineated above. These results indicated that *Broussonetia papyrifera* bark contains significantly high phenolic content.  Table 2 reveals that the constituents were different types of chlorogenic acids (73.99%) including chlorogenic acid (52.22%); the others include isoquercetrin (4.34%) and *broussonetine* A and C (3.67%). Phenolic phytochemicals isolated from PBPs and their structures are shown in Figure 2.

### 3.2. Proliferation Inhibition and Apoptosis of HepG2 Cells Induced by PBPs

**Figure 3:**
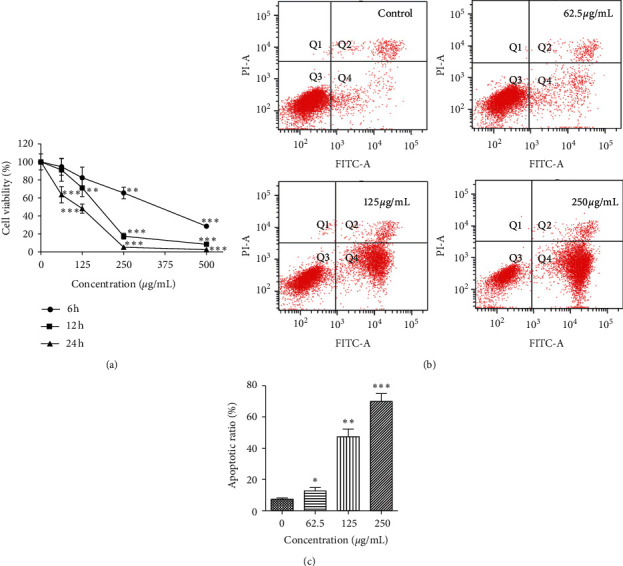
Cell viability of HepG2 cells treated with PBPs. (a) HepG2 cells were treated with PBPs at 62.5, 125, 250, and 500 *μ*g/mL for 6 h, 12 h, and 24 h, respectively (^*∗*^*P* < 0.05/^*∗∗*^*P* < 0.01 versus control); (b) flow cytometry analysis of apoptosis of HepG2 cells after PBPs treatment; (c) the effect of PBPs on apoptotic ratio in HepG2 cells (^*∗*^*P* < 0.05/^*∗∗*^*P* < 0.01/^*∗∗∗*^*P* < 0.001 versus control).

The effect of PBPs on HepG2 cell viability was detected by MTT assay. It was found that the proliferation of HepG2 cells was significantly inhibited by PBPs in a dose-dependent and time-dependent manner (Figure 3(a)). Moreover, 500 *μ*g/mL PBPs exhibited obvious cytotoxicity. Compared with the control group, the viability of HepG2 cells treated by 500 *μ*g/mL PBPs for 6 h was only 28.57%. And, it was 63.48%, 48.11%, and 20.17%, respectively, when exposed to 62.5, 125, and 250 *μ*g/mL PBPs for 24 h. Compared with the control group, the difference of cell viability was statistically significant. Nevertheless, high concentration of PBPs contributed to the necrosis of HepG2 cells.

Flow cytometry analysis was performed to detect apoptosis of HepG2 cells treated with PBPs by Annexin FITC/PI apoptosis detection kit. The ratios of necrosis cells, normal cells, early-apoptosis cells, and late-apoptosis cells are shown in the four-quadrant diagram. Apoptosis of HepG2 cells was induced by PBPs and increased with PBP dose. The early and late-apoptosis ratios of HepG2 cells were 13.6%, 47.4%, and 70%, respectively, corresponding, to 62.5, 125, and 250 *μ*g/mL of PBP treatment for 24 h. HepG2 cells showed various degrees of apoptosis with increase in the PBP concentration (Figure 3(b)). These results indicated that PBPs induced apoptosis of HepG2 cells in a dose-dependent manner (Figure 3(c)).

### 3.3. Effect of PBPs on Redox Balance in HepG2 Cells

**Figure 4:**
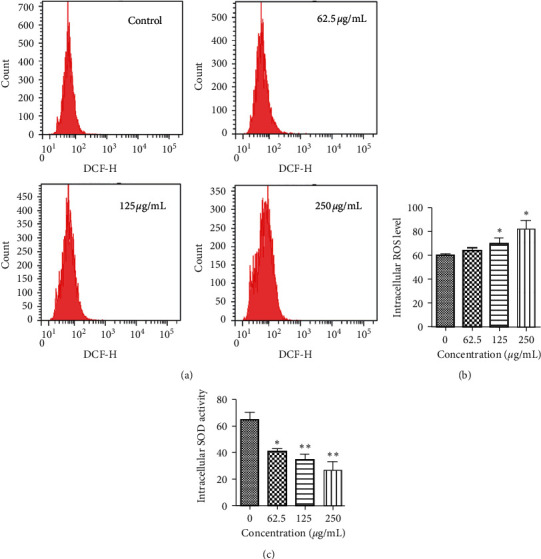
The effect of PBPs on redox balance in HepG2 cells: (a) flow cytometry analysis of intracellular ROS in HepG2 cells after PBPs treatment; (b) the effect of PBPs on intracellular ROS production in HepG2 cells (^*∗*^*P* < 0.05 versus control); (c) the effect of PBPs on intracellular SOD activity in HepG2 cells (^*∗*^*P* < 0.05/ ^*∗∗*^*P* < 0.01 versus control).

Intracellular redox homeostasis is of great importance to the proliferation of cells. We evaluated the redox state of HepG2 cells before and after PBPs exposure. Compared with the control group, HepG2 cells treated with 62.5 *μ*g/mL for 6 h displayed a slight increase of intracellular ROS content. With PBPs concentration increased, the ROS level increased significantly. HepG2 cells treated with PBPs at 250 *μ*g/mL for 6 h had the highest ROS level, which was remarkably higher than that of the control group (Figure 4(b)), and the activity of intracellular SOD was also significantly inhibited in the PBPs treated HepG2 cells (Figure 4(c)), which implied that apoptosis of HepG2 cells was associated with disruption of intracellular redox balance caused by increased ROS level and decreased antioxidant capacity.

### 3.4. Effect of PBPs on Cell Cycle of HepG2 Cells

**Figure 5:**
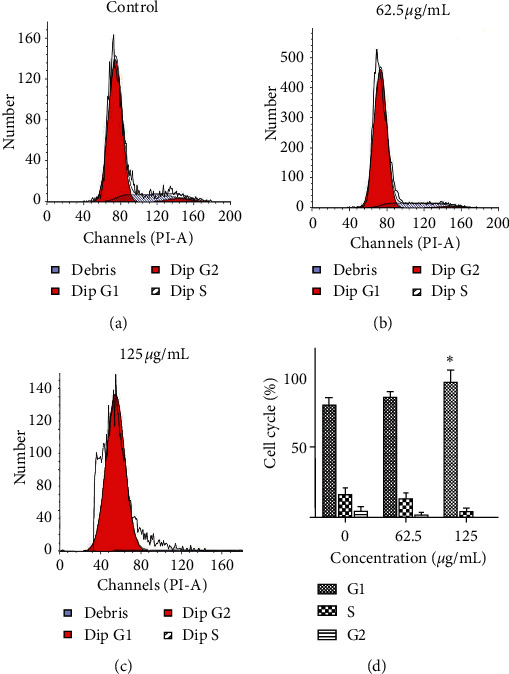
The effect of PBPs on cell cycle of HepG2 cells. The percentage of HepG2 cells at different cell phases treated with 0, 62.5, and 125 *μ*g/mL PBPs for 24 h. The cells arrested at G1 cycle were significantly higher compared with control (^*∗*^*P* < 0.05 versus control).

The effect of PBPs on cycle of HepG2 cells was detected by flow cytometry analysis. HepG2 cells were treated with 62.5 *μ*g/mL PBPs for 24 h, the cell percentage at G1 phase was 85.34%, when treated with 125 *μ*g/mL PBPs, and the percentage came to 95.96%, which was significantly higher than that of control. And, the cell ratios at S and G2 phase were accordingly decreased, which meant that the cell cycle of PBPs treated HepG2 cells was arrested at G1 phase (Figure 5).

### 3.5. Regulation of Apoptosis-Related mRNA Expression in HepG2 Cells Treated by PBPs

**Figure 6:**
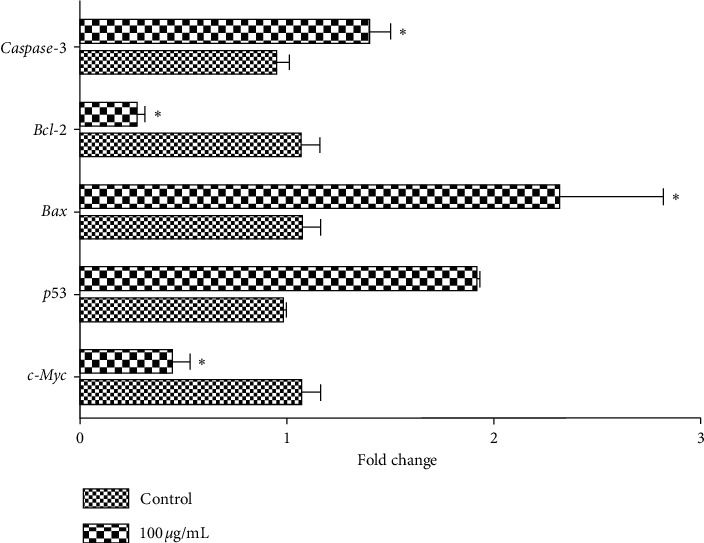
The effect of PBPs on expression levels of apoptosis-related genes in HepG2 cells. The expression of apoptosis-related genes *bax* and *caspase*-3 and tumor suppressor gene *p*53 was significantly upregulated, and the antiapoptosis genes *bcl*-2 and *c-Myc* were significantly downregulated (^*∗*^*P* < 0.05 versus control).

Quantitative real-time PCR was utilized to analyze the expression levels of apoptosis markers genes (e.g., *bax*, *bcl*-2, *p*53, *c-Myc*, and *caspase*-3) in HepG2 cells which were exposed to PBPs at 100 *μ*g/mL for 12 h. The results revealed that expression levels of apoptosis-related genes were significantly altered in HepG2 cells due to PBPs exposure. Expression levels of proapoptosis genes, *bax*, *caspase*-3, and tumor suppressor gene *p*53 were significantly upregulated; meanwhile, expression levels of antiapoptosis genes *bcl*-2 and *c-Myc* were significantly downregulated (Figure 6).

### 3.6. Mitochondria-Mediated Apoptosis of HepG2 Cells Induced by PBPs

**Figure 7:**
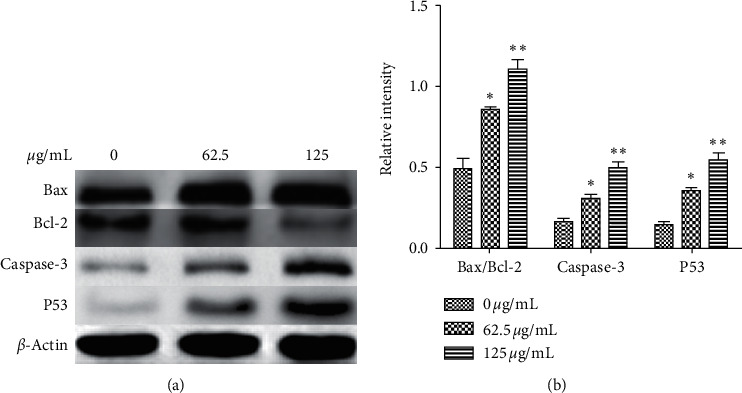
(a) Western blotting detection of mitochondria-mediated apoptosis-related proteins: *Bax*, *Bcl*-2, *Caspase*-3, and *p*53 expression levels; (b) analysis of *Bax*/*Bcl*-2 ratio, *Caspase*-3, and *p*53 expression in HepG2 cells treated with PBPs of 0, 62.5, and 125 *μ*g/mL, respectively (^*∗*^*P* < 0.05/^*∗∗*^*P* < 0.01 versus control).

It is well-known that the changes of *Bax*, *Bcl*-2, *Caspase*-3, and *p*53 expression and *Bax*/*Bcl*-2 ratio are important features of the mitochondria-mediated apoptosis pathway. In order to confirm exactly the apoptosis pathway of HepG2 cells caused by PBPs, HepG2 cells were treated with 62.5 and 125 *μ*g/mL of PBPs for 24 h and protein expression levels of *Bax*, *Bcl*-2, *Caspase*-3, and *p*53 were determined by western blotting. The results demonstrated that *Bax*, *Caspase*-3, *p*53, and *Bax*/*Bcl*-2 ratio in HepG2 cells exposed to PBPs were significantly increased with a dose-dependent manner (Figure 7).

### 3.7. Effect of PBPs on ERK and AKT Phosphorylation in HepG2 Cells

**Figure 8:**
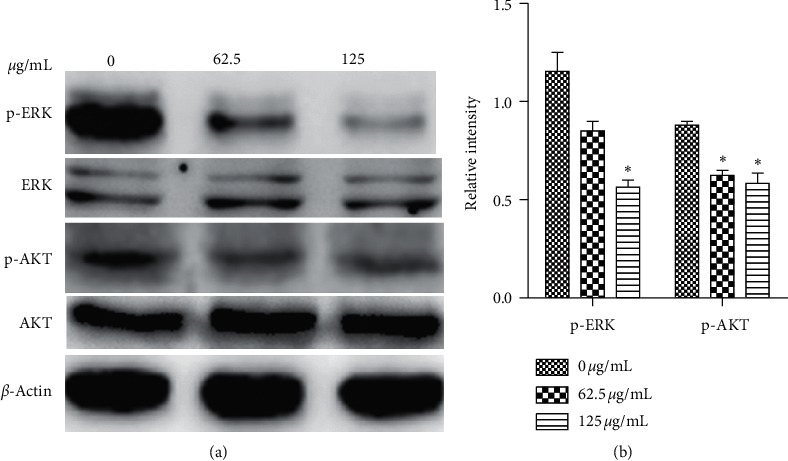
The effect of PBPs on phosphorylation of ERK and AKT in HepG2 cells: (a) western blotting detection of ERK, p-ERK, AKT, and p-AKT expression levels, (b) relative levels of p-ERK and p-AKT in HepG2 cells treated with PBPs of 0, 62.5, and 125 *μ*g/mL, respectively (^*∗*^*P* < 0.05 versus control).

To further explore the mechanism of mitochondria-mediated apoptosis induced by PBPs, we presumed the possible roles of ERK and AKT in the process of apoptosis of the PBPs treated HepG2 cells. The results indicated that ERK and AKT expression of the cells were not affected, while, the p-ERK and p-AKT expression, which were important for the proliferation and survival of HepG2 cells, were markedly reduced (Figure 8). It could be reasonably confirm that apoptosis of HepG2 cells induced by PBPs was associated with inhibition of ERK and PI-3K/AKT signaling pathways.

## 4. Discussion

PBPs, extracted from *Broussonetia papyrifera* bark, mainly consisted of chlorogenic acid, cryptochlorogenic acid, and cis-5-coffee acylchlorogenic acid, followed by a small quantity of 3, 4, 5-trimethoxyphenyl-1-O-*β*-D-glucopyranoside, isoquercetin, and 4, 5-dicaffeoylquinic acid. PBPs inhibited the proliferation of HepG2 cells, and the cell cycle was blocked in the G1 phase. Huang et al. [20] also reported the similar results upon the treatment of HepG2 cells with isoquercetrin. Importantly, some studies found that the growth of HepG2 cells could be inhibited by chlorogenic acid [21], and the relatively effective concentration was similar to our data. However, the effect of chlorogenic acid on HepG2 cells proliferation was controversial. Miccadei et al. [22] claimed that artichoke extracts (chlorogenic acid and two dicaffeoylquinic acids) had a proapoptotic activity in HepG2 cells. On the contrary, Granado-Serrano et al. [23] found that ROS production of chlorogenic acid treated HepG2 cells was decreased, but no prominent effect on apoptosis was observed. It is speculated that chlorogenic acid might exert antitumor activity through different mechanism instead of inducing HepG2 cells apoptosis directly. Moreover, the total alkaloids from *Broussonetia papyrifera* fruits exhibited lower cytotoxicity on normal human skin epidermal cells than that of certain cancer lines [24]. Except for chlorogenic acid, the role of other PBPs components in inducing apoptosis remains unclear; we assumed that chlorogenic acid might have a synergistic role with other active constituents of PBPs.

*Bcl*-2 family members consist of proapoptosis proteins (*Bax*, Noxa, Puma, Bim, and Bid), and antiapoptosis proteins (*Bcl*-2 and *Bcl*-xl) play a crucial role in controlling the mitochondrial pathway of apoptosis [25, 26]. With the *Bax*/*Bcl*-2 protein ratio increase, the structure and permeability of the mitochondrial permeability transition pore (mPTP) change, ultimately leading to the mitochondrial-driven collapse [27]. In this study, *Bax* expression was significantly upregulated in PBP-treated HepG2 cells, whereas *Bcl*-2 was downregulated simultaneously. Moreover, *Caspase*-3 was also significantly upregulated. Therefore, the downregulation of *Bcl*-2 and upregulation of Bax may contribute to PBPs-induced HepG2 cells apoptosis. We concluded that PBPs inhibited the proliferation of HepG2 cells through the mitochondria-mediated apoptosis pathway.

*p*53 is known to induce apoptosis and plays an important role in mitochondria-mediated apoptosis [28]. In this study, the cellular cycle of PBP-treated HepG2 cells (*p*53 wild-type) was arrested at G1 phase and accompanied by *p*53 expression upregulation and *c-Myc* expression downregulation; it could be inferred that *p*53 and *C-Myc* played vital roles in the apoptosis process of HepG2 cells induced by PBPs.

Mitochondrion is one of the major sources of ROS. Mitochondrion dysfunction contributes to more ROS production and causes mitochondria-mediated apoptosis, leading to a vicious circle [29]. Tumor cells with a higher level of ROS tend to be killed more easily than normal ones with a lower level of ROS [30]. In the present work, the production of intracellular ROS of the PBP-treated HepG2 cells was increased significantly in a dose-dependent manner. Mitochondrion is considered is the major source of ROS, which implied that mitochondrial dysfunction was due to PBP treatment. Therefore, it is promising to screen new anticancer drugs from natural compounds which have the potential to induce ROS production in tumor cells [31]. SOD, known to be an effective antioxidant, plays an important role in eliminating peroxides and free radicals produced in the metabolic process of cells in vivo and maintaining cellular redox homeostasis in a variety of tissues [32]. Our finding showed that the intracellular SOD activity of PBP-treated HepG2 cells was significantly deceased, and the imbalance of cellular redox status was aggravated, which resulted in the apoptosis of HepG2 cells.

The sustained activation of ERK, acting as an important signaling molecule in cell proliferation [33, 34], is also necessary for cancer cells survival and proliferation [35]. The inhibition of ERK contributes to the increased occurrence of cell death. As an important channel in downstream of c-Met (oncogene), ERK is required for the phosphorylation of Bad, an antiapoptotic protein of *Bcl*-2 family [36, 37]. ERK inhibitor was suggested as a potential antitumor agent [38]. Our findings showed that ERK phosphorylation in HepG2 cells treated by PBPs was significantly inhibited in a dose-dependent manner, which supported the notion that the downregulation of ERK activity was beneficial to HCC treatment.

The PI-3K/AKT signaling pathway is important for tumor cells growth, and the phosphorylation of AKT directly blocks a variety of downstream targets, such as the proapoptotic proteins Bad, *Bax*, and *Caspase*-9 [39]. Inhibiting AKT phosphorylation has been proved as a novel target for therapeutic agents in human cancer [40]. Therefore, it could be concluded that the decreased phosphorylated AKT induced by PBPs might contribute to the promotion of apoptosis by multiple targets.

## 5. Conclusions

In summary, this study illustrates that PBPs induce mitochondria-mediated apoptosis of HepG2 cells via the inactivation of ERK and AKT signaling pathways. Our research findings can be applied towards characterization and development of new antitumor dugs from *Broussonetia papyrifera* extracts.

## Figures and Tables

**Table 1:** Sequences of primers used for quantitative real-time PCR.

Gene	Forward	Reverse	bp
*Bax*	TGCTTCAGGGTTTCATCCAG	GGCGGCAATCATCCTCTG	170
*Bcl*-2	AGGAAGTGAACATTTCGGTGAC	GCTCAGTTCCAGGACCAGGC	146
*p*53	CCCAGCCAAAGAAGAAACCA	TTCCAAGGCCTCATTCAGCT	101
*c-Myc*	TGAACACAGCGAATGTTTCC	TTAGGAGCGCTCAGGTCTGT	69
*Caspase*-3	ACATGGCGTGTCATAAAATACC	CACAAAGCGACTGGATGAAC	120
*β-actin*	TCACCCACACTGTGCCCATCTACGA	AGCGGAACCGCTCATTGCCAATGG	294

**Table 2:** Characterization of PBP.

Peak	*t*_*R*_ (min)	Tentative compounds	Peak area ratio (%)	Main MS^2^ (m/z)	[M-H]^−^ (m/z)
*P*1	8.22	Chlorogenic acid	52.22	191	353.0872
*P*2	8.99	Cryptochlorogenic acid	8.28	135, 173, 179, 191	353.0874
*P*3	9.40	3,4,5-Trimethoxyphenyl-1-O-*β*-D-xylopyranosyl-*β*-D-glucopyranoside	2.31	293, 233, 161, 153, 89, 59	477.1605
*P*4	10.82	*cis*-form-5-coffee acylchlorogenic acid	8.62	191	353.0875
*P*5	17.17	Isoquercetin	4.34	271, 255	463.0874
*P*6	21.1	4,5-Dicaffeoylquinic acid	2.83	353, 191, 179, 173	515.1193

## Data Availability

The data used to support the findings of this study are available from the corresponding author upon request.

## Abbreviations

DMEM: Dulbecco's modified eagle medium
DCFH-DA: 2,7-dichlorofluorescin diacetate
ERK: Extracellular signal-regulated kinase
HCC: Hepatocellular carcinoma
HRP: Horseradish peroxidase
FBS: Fetal bovine serum
FITC: Fluorescein isothiocyanate
mPTP: Mitochondrial permeability transition pore
MTT: 3-(4,5-Dimethylthiazol-2-yl)-2,5-diphenyltetrazolium bromide
MS: Mass spectrometry
PBPs: Polyphenols from *Broussonetia papyrifera*
PBS: Phosphate-buffered saline
PI: Propidium iodide
ROS: Reactive oxygen species
SOD: Superoxide dismutase
UPLC: Ultra-high-performance liquid chromatography.

## References

[1] Han Q., Zhao H., Jiang Y., Yin C., Zhang J. (2019). HCC-derived exosomes: critical player and target for cancer immune escape. *Cells*.

[2] Singal A. G., El-Serag H. B. (2015). Hepatocellular carcinoma from epidemiology to prevention: translating knowledge into practice. *Clinical Gastroenterology and Hepatology*.

[3] Yang J. D., Roberts L. R. (2010). Epidemiology and management of hepatocellular carcinoma. *Infectious Disease Clinics of North America*.

[4] Yarchoan M., Agarwal P., Villanueva A. (2019). Recent developments and therapeutic strategies against hepatocellular carcinoma. *Cancer Research*.

[5] Li S., Yuan S., Zhao Q., Wang B., Wang X., Li K. (2018). Quercetin enhances chemotherapeutic effect of doxorubicin against human breast cancer cells while reducing toxic side effects of it. *Biomedicine & Pharmacotherapy*.

[6] Hsu H.-H., Chen M.-C., Baskaran R. (2018). Oxaliplatin resistance in colorectal cancer cells is mediated via activation of ABCG2 to alleviate ER stress induced apoptosis. *Journal of Cellular Physiology*.

[7] Dutta S., Mahalanobish S., Saha S., Ghosh S., Sil P. C. (2019). Natural products: an upcoming therapeutic approach to cancer. *Food and Chemical Toxicology*.

[8] Dongjoo L., Kim I. Y., Saha S., Choi K. S. (2016). Paraptosis in the anti-cancer arsenal of natural products. *Pharmacology & Therapeutics*.

[9] Barker C. (2002). Plate 432. Broussonetia papyrifera. *Curtis’s & Apos s Botanical Magazine*.

[10] Sohn H.-Y., Kwon C. S., Son K. H. (2010). Fungicidal effect of prenylated flavonol, papyriflavonol A, isolated from *Broussonetia papyrifera* (L.) vent. against Candida albicans. *Journal of Microbiology and Biotechnology*.

[11] Xu M.-L., Wang L., Hu J.-H., Lee S. K., Wang M.-H. (2010). Antioxidant activities and related polyphenolic constituents of the methanol extract fractions from Broussonetia papyrifera stem bark and wood. *Food Science and Biotechnology*.

[12] Lin L.-W., Chen H.-Y., Wu C.-R. (2008). Comparison with various parts ofBroussonetia papyriferaas to the antinociceptive and anti-inflammatory activities in rodents. *Bioscience, Biotechnology, and Biochemistry*.

[13] Guo M. X., Wang M., Deng H., Zhang X., Wang Z.-Y. (2013). A novel anticancer agent Broussoflavonol B downregulates estrogen receptor (ER)-*α*36 expression and inhibits growth of ER-negative breast cancer MDA-MB-231 cells. *European Journal of Pharmacology*.

[14] Naveen Kumar N., Ramakrishnaiah H., Krishna V., Radhika M. (2014). Cytotoxic activity of Broussonetia papyrifera (I.) vent on MCF-7, HeLa and HepG2 cell lines. *International Journal of Pharmacy and Pharmaceutical Sciences*.

[15] Wang L., Son H. J., Xu M.-L., Hu J.-H., Wang M.-H. (2010). Anti-inflammatory and anticancer properties of dichloromethane and butanol fractions from the stem bark of Broussonetia papyrifera. *Journal of the Korean Society for Applied Biological Chemistry*.

[16] Li H., Chen J., Xiong C., Wei H., Yin C., Ruan J. (2014). Apoptosis induction by the total flavonoids from *Arachniodes exilis* in HepG2 cells through reactive oxygen species-mediated mitochondrial dysfunction involving MAPK activation. *Evidence-based Complementary & Alternative Medicine*.

[17] Min L., He B., Hui L. (2011). Mitogen-activated protein kinases in hepatocellular carcinoma development. *Seminars in Cancer Biology*.

[18] Zhang L., Tu Y., He W., Peng Y., Qiu Z. (2015). A novel mechanism of hepatocellular carcinoma cell apoptosis induced by lupeol via brain-derived neurotrophic factor inhibition and glycogen synthase kinase 3 beta reactivation. *European Journal of Pharmacology*.

[19] Ahamed M., Akhtar M. J., Siddiqui M. A. (2011). Oxidative stress mediated apoptosis induced by nickel ferrite nanoparticles in cultured A549 cells. *Toxicology*.

[20] Huang G., Tang B., Tang K. (2014). Isoquercitrin inhibits the progression of liver cancer in vivo and in vitro via the MAPK signalling pathway. *Oncology Reports*.

[21] Yan Y., Liu N., Hou N., Dong L., Li J. (2017). Chlorogenic acid inhibits hepatocellular carcinoma in vitro and in vivo. *The Journal of Nutritional Biochemistry*.

[22] Miccadei S., Venere D. D., Cardinali A. (2008). Antioxidative and apoptotic properties of polyphenolic extracts from edible part of Artichoke (*Cynara scolymus* L.) on cultured rat hepatocytes and on human hepatoma cells. *Nutrition and Cancer*.

[23] Granado-Serrano A. B., Angeles Martín M., Izquierdo-Pulido M., Goya L., Bravo L., Ramos S. (2007). Molecular mechanisms of (−)−epicatechin and chlorogenic acid on the regulation of the apoptotic and survival/proliferation pathways in a human hepatoma cell line. *Journal of Agricultural and Food Chemistry*.

[24] Pang S. Q., Wang G.-Q., Lin J.-S., Diao Y., Xu R.-A. (2014). Cytotoxic activity of the alkaloids from Broussonetia papyrifera fruits. *Pharmaceutical Biology*.

[25] Wei M. C. (2004). Bcl-2-related genes in lymphoid neoplasia. *International Journal of Hematology*.

[26] Chen Q., Lesnefsky E. J. (2011). Blockade of electron transport during ischemia preserves bcl-2 and inhibits opening of the mitochondrial permeability transition pore. *FEBS Letters*.

[27] Sawada M., Nakashima S., Banno Y. (2000). Ordering of ceramide formation, caspase activation, and Bax/Bcl-2 expression during etoposide-induced apoptosis in C6 glioma cells. *Cell Death & Differentiation*.

[28] Wickramasekera N. T., Das G. M. (2014). Tumor suppressor P53 and estrogen receptors in nuclear–mitochondrial communication. *Mitochondrion*.

[29] Ito K., Hirao A., Arai F. (2006). Erratum: reactive oxygen species act through p38 MAPK to limit the lifespan of hematopoietic stem cells. *Nature Medicine*.

[30] Paz-Elizur T., Sevilya Z., Leitner-Dagan Y., Elinger D., Roisman L. C., Livneh Z. (2008). DNA repair of oxidative DNA damage in human carcinogenesis: potential application for cancer risk assessment and prevention. *Cancer Letters*.

[31] Trachootham D., Zhou Y., Zhang H. (2006). Selective killing of oncogenically transformed cells through a ROS-mediated mechanism by *β*-phenylethyl isothiocyanate. *Cancer Cell*.

[32] Tahir A. (2015). Imbalance of serum trace elements in acute leukemia contributes to pro and anti-oxidative stress via ROS and SOD regulation: a population based study. *International Endodontic Journal*.

[33] Chang L., Karin M. (2001). Mammalian MAP kinase signalling cascades. *Nature*.

[34] Fang X., Yu S., Eder A. (1999). Regulation of BAD phosphorylation at serine 112 by the Ras-mitogen-activated protein kinase pathway. *Oncogene*.

[35] Ballif B. A., Blenis J. (2001). Molecular mechanisms mediating mammalian mitogen-activated protein kinase (MAPK) kinase (MEK)-MAPK cell survival signals. *Cell Growth & Differentiation*.

[36] Wang J., Gui Z., Deng L. (2012). c-Met upregulates aquaporin 3 expression in human gastric carcinoma cells via the ERK signalling pathway. *Cancer Letters*.

[37] Park J. H., Zhao T. T., Park K. H., Lee M. K. (2019). Repeated treatments with the D1 dopamine receptor agonist SKF-38393 modulate cell viability via sustained ERK-Bad-Bax activation in dopaminergic neuronal cells. *Behavioural Brain Research*.

[38] O”Neil B. H., Goff L. W., Kauh J. S. W. (2011). Phase II study of the mitogen-activated protein kinase 1/2 inhibitor selumetinib in patients with advanced hepatocellular carcinoma. *Journal of Clinical Oncology*.

[39] Yuan L., Wang J., Xiao H., Xiao C., Wang Y., Liu X. (2012). Isoorientin induces apoptosis through mitochondrial dysfunction and inhibition of PI3K/Akt signaling pathway in HepG2 cancer cells. *Toxicology and Applied Pharmacology*.

[40] Franke T. F. (2008). PI3K/Akt: getting it right matters. *Oncogene*.

